# Supplementary material for: First COVID-19 Booster Dose in the General Population: A Systematic Review and Meta-Analysis of Willingness and Its Predictors
Source: Vaccines (Basel). 2022 Jul 8;10(7):1097. doi: 10.3390/vaccines10071097 (PMC9323526; doi:10.3390/vaccines10071097)
Supplement: Supplementary file 1 [file vaccines-10-01097-s001.zip › Supplementary Table S1.pdf]

Supplement Table S1. Quality of studies included in this systematic review.

	(Lounis et al., 2022)	(Rzymski et al., 2021)	(Lai et al., 2021)	(Al-Qerem et al., 2022)	(Wu et al., 2022)	(Miao et al., 2022)
1. Were the criteria for inclusion in the sample clearly defined?	Yes	Yes	Yes	Yes	Yes	Yes
2. Were the study subjects and the setting described in detail?	Yes	Yes	Yes	Yes	Yes	Yes
3. Was the exposure measured in a valid and reliable way?	Yes	Yes	Yes	Yes	Yes	Yes
4. Were objective, standard criteria used for measurement of the condition?	Yes	Yes	Yes	Yes	Yes	Yes
5. Were confounding factors identified?	Yes	No	Yes	Yes	Yes	Yes
6. Were strategies to deal with confounding factors stated?	Yes	No	Yes	Yes	Yes	Yes
7. Were the outcomes measured in a valid and reliable way?	Yes	Yes	Yes	Yes	Yes	Yes
8. Was appropriate statistical analysis used?	Yes	Yes	Yes	Yes	Yes	Yes
**Risk of bias**	**Low**	**Moderate**	**Low**	**Low**	**Low**	**Low**

NA: not applicable

Supplement Table S1 (continued). Quality of studies included in this systematic review.

	(Chu et al., 2022)	(Jørgensen et al., 2022)	(Wang et al., 2022)	(Rababa'h et al., 2021)	(Paul & Fancourt, 2022)	(Yadete et al., 2021)	(Yoshida et al., 2022)	(Wirawan et al., 2022)
1. Were the criteria for inclusion in the sample clearly defined?	Yes	Yes	Yes	Yes	Yes	Yes	Yes	Yes
2. Were the study subjects and the setting described in detail?	Yes	Yes	Yes	Yes	Yes	Yes	Yes	Yes
3. Was the exposure measured in a valid and reliable way?	Yes	Yes	Yes	Yes	Yes	Yes	Yes	Yes
4. Were objective, standard criteria used for measurement of the condition?	Yes	Yes	Yes	Yes	Yes	Yes	Yes	Yes
5. Were confounding factors identified?	Yes	Yes	Yes	NA	Yes	NA	Yes	Yes
6. Were strategies to deal with confounding factors stated?	Yes	Yes	Yes	NA	Yes	NA	Yes	Yes
7. Were the outcomes measured in a valid and reliable way?	Yes	Yes	Yes	Yes	Yes	Yes	Yes	Yes
8. Was appropriate statistical analysis used?	Yes	Yes	Yes	Yes	Yes	Yes	Yes	Yes
**Risk of bias**	**Low**	**Low**	**Low**	**Low**	**Low**	**Low**	**Low**	**Low**

NA: not applicable

References

Al-Qerem, W., Al Bawab, A. Q., Hammad, A., Ling, J., & Alasmari, F. (2022). Willingness of the Jordanian Population to Receive a COVID-19 Booster Dose: A Cross-Sectional Study. *Vaccines*, *10*(3), 410. https://doi.org/10.3390/vaccines10030410

Chu, D.-T., Vu Thi, H., Nguyen Thi, Y. V., Nguyen, M.-A., Bui, N.-L., Hoang, V. T., Hoang Nam, D., Do, D.-L., than, V. T., & Al-Tawfiq, J. A. (2022). Willingness to receive COVID-19 vaccine booster doses for adults and their children in Vietnam. *Journal of Human Behavior in the Social Environment*, 1–13. https://doi.org/10.1080/10911359.2022.2046235

Jørgensen, F. J., Nielsen, L. H., & Petersen, M. B. (2022). Willingness to Take the Booster Vaccine in a Nationally Representative Sample of Danes. *Vaccines*, *10*(3), 425. https://doi.org/10.3390/vaccines10030425

Lai, X., Zhu, H., Wang, J., Huang, Y., Jing, R., Lyu, Y., Zhang, H., Feng, H., Guo, J., & Fang, H. (2021). Public Perceptions and Acceptance of COVID-19 Booster Vaccination in China: A Cross-Sectional Study. *Vaccines*, *9*(12), 1461. https://doi.org/10.3390/vaccines9121461

Lounis, M., Bencherit, D., Rais, M. A., & Riad, A. (2022). COVID-19 Vaccine Booster Hesitancy (VBH) and Its Drivers in Algeria: National Cross-Sectional Survey-Based Study. *Vaccines*, *10*(4), 621. https://doi.org/10.3390/vaccines10040621

Miao, Y., Li, Y., Zhang, W., Wu, J., Gu, J., Wang, M., Wei, W., Ye, B., Miao, C., Tarimo, C. S., & Dong, W. (2022). The Psychological Experience of COVID-19 Vaccination and Its Impact on the Willingness to Receive Booster Vaccines among the Chinese Population: Evidence from a National Cross-Sectional Study. *International Journal of Environmental Research and Public Health*, *19*(9), 5464. https://doi.org/10.3390/ijerph19095464

Paul, E., & Fancourt, D. (2022). Predictors of uncertainty and unwillingness to receive the COVID-19 booster vaccine: An observational study of 22,139 fully vaccinated adults in the UK. *The Lancet Regional Health - Europe*, *14*, 100317. https://doi.org/10.1016/j.lanepe.2022.100317

Rababa'h, A. M., Abedalqader, N. N., & Ababneh, M. (2021). Jordanians' willingness to receive heterologous prime-boost COVID-19 vaccination and vaccine boosters. *European Review for Medical and Pharmacological Sciences*, *25*(23), 7516–7525. https://doi.org/10.26355/eurrev_202112_27452

Rzymski, P., Poniedziałek, B., & Fal, A. (2021). Willingness to Receive the Booster COVID-19 Vaccine Dose in Poland. *Vaccines*, *9*(11), 1286. https://doi.org/10.3390/vaccines9111286

Wang, X., Liu, L., Pei, M., Li, X., Li, N., Research Center of Clinical Epidemiology, Peking University Third Hospital, Beijing, China, & Department of Infectious Diseases, Peking University Third Hospital, Beijing, China. (2022). Willingness of the General Public to Receive A COVID-19 Vaccine Booster—China, April–May 2021. *China CDC Weekly*, *4*(4), 66–70. https://doi.org/10.46234/ccdcw2022.013

Wirawan, G. B. S., Harjana, N. P. A., Nugrahani, N. W., & Januraga, P. P. (2022). Health Beliefs and Socioeconomic Determinants of COVID-19 Booster Vaccine Acceptance: An Indonesian Cross-Sectional Study. *Vaccines*, *10*(5), 724. https://doi.org/10.3390/vaccines10050724

Wu, F., Yuan, Y., Deng, Z., Yin, D., Shen, Q., Zeng, J., Xie, Y., Xu, M., Yang, M., Jiang, S., Zhang, C., Lu, H., & Sun, C. (2022). Acceptance of COVID-19 booster vaccination based on the protection motivation theory: A cross-sectional study in China. *Journal of Medical Virology*, jmv.27825. https://doi.org/10.1002/jmv.27825

Yadete, T., Batra, K., Netski, D. M., Antonio, S., Patros, M. J., & Bester, J. C. (2021). Assessing Acceptability of COVID-19 Vaccine Booster Dose among Adult Americans: A Cross-Sectional Study. *Vaccines*, *9*(12), 1424. https://doi.org/10.3390/vaccines9121424

Yoshida, M., Kobashi, Y., Kawamura, T., Shimazu, Y., Nishikawa, Y., Omata, F., Zhao, T., Yamamoto, C., Kaneko, Y., Nakayama, A., Takita, M., Ito, N., Kawashima, M., Sugiura, S., Shibuya, K., Iwami, S., Kim, K., Iwanami, S., Kodama, T., & Tsubokura, M. (2022). Factors Associated with COVID-19 Vaccine Booster Hesitancy: A Retrospective Cohort Study, Fukushima Vaccination Community Survey. *Vaccines*, *10*(4), 515. https://doi.org/10.3390/vaccines10040515
