# Supplementary figures and images for: First COVID-19 Booster Dose in the General Population: A Systematic Review and Meta-Analysis of Willingness and Its Predictors
Source: Vaccines (Basel). 2022 Jul 8;10(7):1097. doi: 10.3390/vaccines10071097 (PMC9323526; doi:10.3390/vaccines10071097)

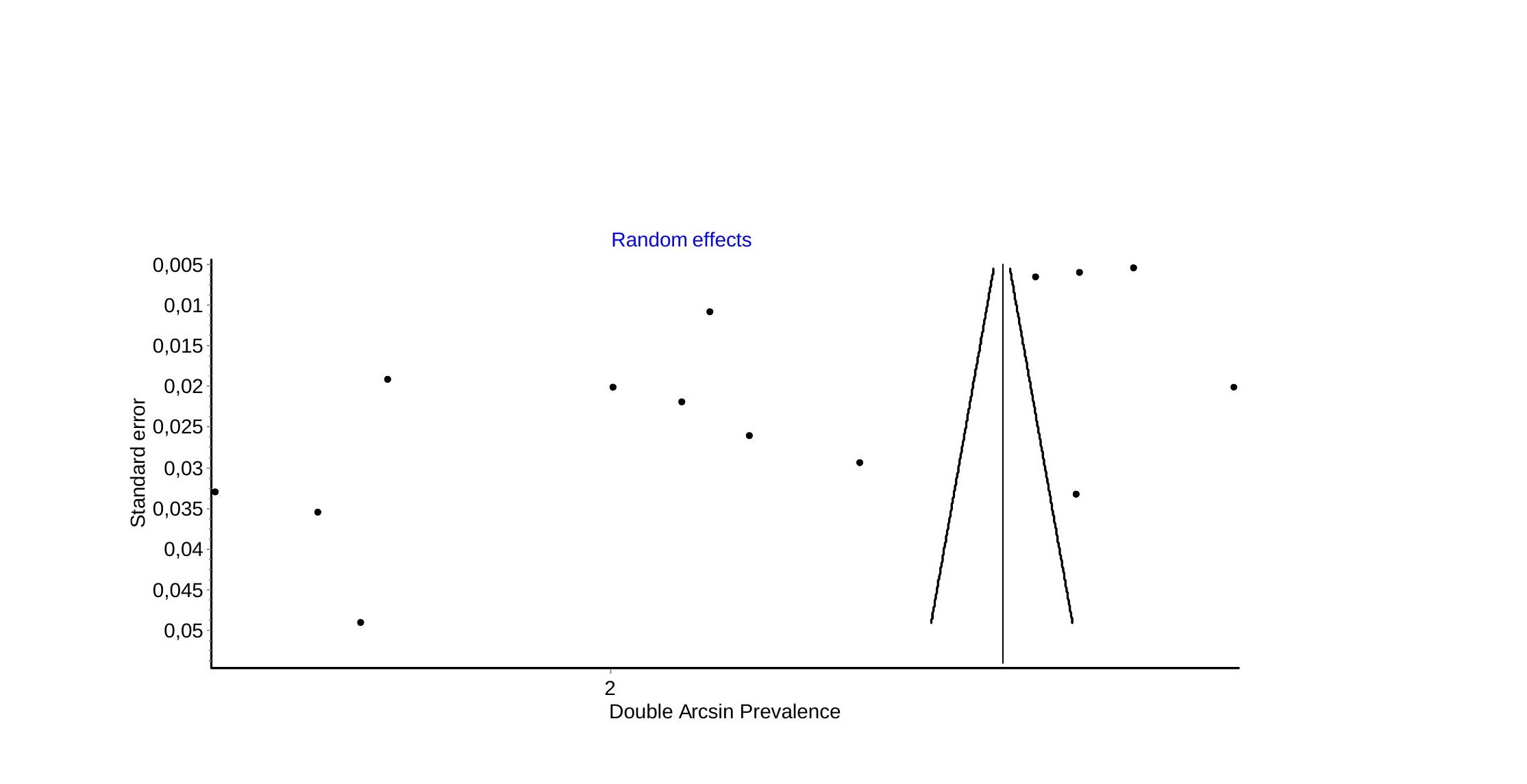

Supplement: Supplementary file 1 [file vaccines-10-01097-s001.zip › supplementary figure S1.jpg]

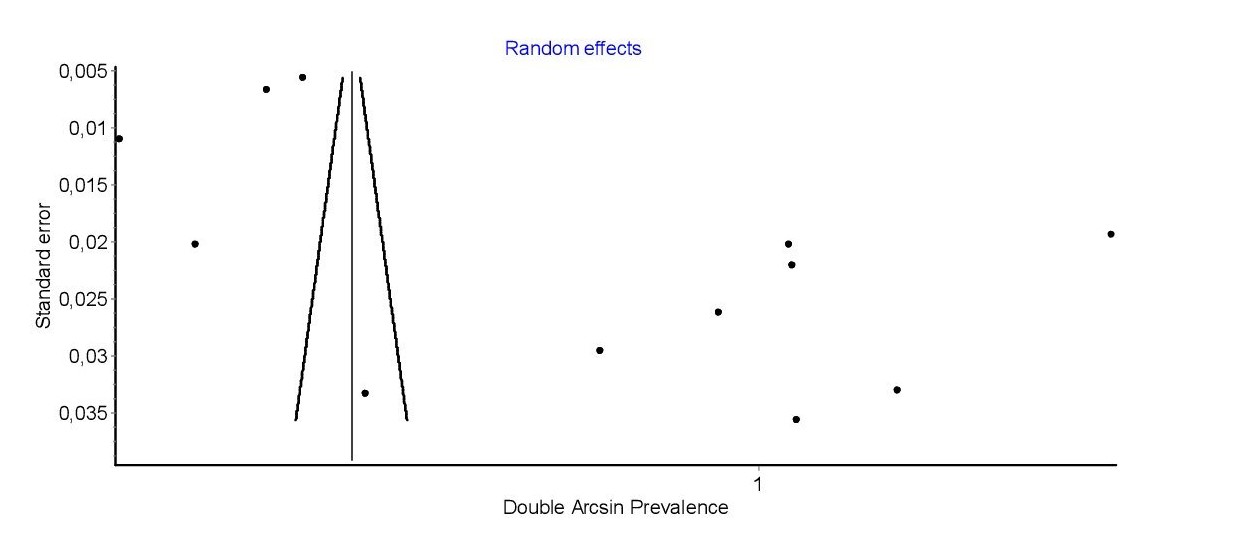

Supplement: Supplementary file 1 [file vaccines-10-01097-s001.zip › Supplementary Figure S2.jpg]

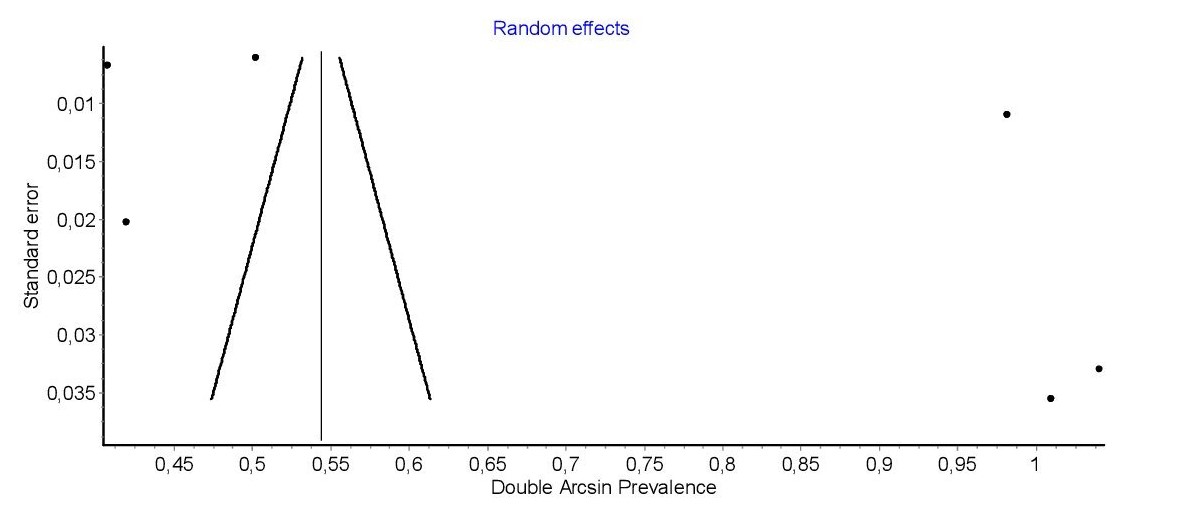

Supplement: Supplementary file 1 [file vaccines-10-01097-s001.zip › supplementary figure S3.jpg]
